# 来那度胺联合硼替佐米和地塞米松诱导治疗初诊多发性骨髓瘤患者的疗效和安全性

**DOI:** 10.3760/cma.j.issn.0253-2727.2022.08.007

**Published:** 2022-08

**Authors:** 卫芹 姚, 灵芝 颜, 京晶 商, 松 金, 晓兰 施, 霜 颜, 治 严, 晴晴 王, 琤琤 傅, 德沛 吴

**Affiliations:** 苏州大学附属第一医院，江苏省血液研究所，苏州 215000 The First Affiliated Hospital of Soochow University, Jiangsu Institute of Hematology, Suzhou 215000, China

**Keywords:** 多发性骨髓瘤, 硼替佐米, 来那度胺, Multiple myeloma, Bortezomib, Lenalidomide

## Abstract

**目的:**

评估来那度胺联合硼替佐米和地塞米松（VRD）治疗初诊多发性骨髓瘤（MM）患者的疗效和安全性。

**方法:**

回顾性分析自2018年11月至2021年2月在苏州大学附属第一医院诊断并接受VRD方案诱导治疗的150例初诊MM患者，评价初诊MM患者应用VRD方案诱导治疗的疗效及安全性。

**结果:**

中位随访时间22（1～38）个月，2例（1.3％）患者治疗早期死亡，148例（98.7％）完成诱导治疗。116例（77.3％）患者动员采集自体造血干细胞，采集物合格者101例（87.1％），其中采集物优秀者48例（41.4％）。3年无进展生存（PFS）率为59％，3年总生存（OS）率为83％。诱导治疗后完全缓解/严格意义的完全缓解率为54.4％，≥非常好的部分缓解率为77.3％，总体反应率为86.0％，微小残留病阴性率为46.0％。细胞遗传学高危患者的疗效与标危患者相比差异无统计学意义（*P*＝0.456），遗传学高危患者的中位PFS时间较标危患者缩短（33个月对未达到，*P*＝0.014），中位OS时间的差异无统计学意义（均未达到，*P*＝0.072）。血液学不良事件中血小板减少发生率最高（72％），其次为中性粒细胞减少（42％）和贫血（20％）。非血液学不良事件中周围神经炎发生率最高（56.7％）；消化道症状主要为便秘（30.0％）和腹泻（17.3％）；感染主要为上呼吸道感染（23.3％）和肺部感染（7.3％）；转氨酶升高发生率较高（32.6％）。与肾功能正常患者相比，初诊时肾功能不全患者血小板减少（90.0％对63.7％，*P*＝0.001）、贫血（33.3％对13.7％，*P*＝0.005）、中性粒细胞减少（54.2％对36.3％，*P*＝0.038）、腹泻（27.1％对12.7％，*P*＝0.030）、肢体水肿（20.8％对3.9％，*P*＝0.030）、发热（20.8％对4.9％，*P*＝0.006）、血栓（8.3％对0，*P*＝0.016）、肾功能恶化（20.8％对3.9％，*P*＝0.030）的发生率较高。

**结论:**

VRD方案治疗初诊MM疗效显著，不影响造血干细胞采集，不良事件可控，但肾功能不全患者不良事件发生率较高。

## Abstract (en)

**Objective:**

This study aimed to evaluate the efficacy and safety of lenalidomide combined with bortezomib and dexamethasone（VRD）in the treatment of newly diagnosed multiple myeloma（MM）.

**Methods:**

A total of 150 newly diagnosed patients with MM diagnosed in The First Affiliated Hospital of Soochow University from November 2018 to February 2021 and received VRD as the induction regimen were included to evaluate the safety and efficacy of VRD induction therapy for newly diagnosed MM.

**Results:**

The median follow-up was 22 months, two patients（1.3％）died early after treatment, and 148 patients（98.7％）completed induction therapy. 116 patients（77.3％）were mobilized to collect autologous hematopoietic stem cells, 101 cases（87.1％）were qualified in the collection, of which 48 cases（41.4％）were excellent in the collection. The 3-year progression-free survival（PFS）rate was 59％, and the 3-year overall survival（OS）rate was 83％. After induction, complete remission（CR）/stringent CR rate was 54.4％, ≥very good partial remission rate was 77.3％, overall response rate was 86.0％, and minimal residual disease negative rate was 46.0％. There was no statistically significant difference in the efficacy of cytogenetic high-risk patients compared with standard risk patients（*P*＝0.456）. The median PFS time of cytogenetic high-risk patients was shorter than that of standard risk patients（not reached *vs* 33 months, *P*＝0.014）. There was no statistically significant difference in the median OS time（not reached *vs* not reached, *P*＝0.072）. The highest incidence of hematological adverse events was thrombocytopenia（72％）, followed by neutropenia（42％）and anemia（20％）. The highest incidence of non-hematological adverse events was peripheral neuritis（56.7％）. The main digestive tract symptoms include constipation（30.0％）and diarrhea（17.3％）. Upper respiratory tract infection（23.3％）and lung infection（7.3％）are the main infections. The incidence of adverse thrombocytopenia（90.0％ *vs* 63.7％, *P*＝0.001）, neutropenia（54.2％ *vs* 36.3％, *P*＝0.038）, anemia（33.3％ *vs* 13.7％, *P*＝0.005）, diarrhea（27.1％ *vs* 12.7％, *P*＝0.030）, limb edema（20.8％ *vs* 3.9％, *P*＝0.030）, fever（20.8％ *vs* 4.9％, *P*＝0.006）, thrombosis（8.3％ *vs* 0, *P*＝0.016）, and renal function deterioration（20.8％ *vs* 3.9％, *P*＝0.030）in patients with renal insufficiency was higher than that in patients with normal renal function.

**Conclusion:**

The VRD regimen has a significant effect on newly diagnosed MM, does not affect the hematopoietic stem cell collection, and has controllable adverse events; however, the incidence of adverse events was higher in patients with renal insufficiency.

近年来，随着蛋白酶体抑制剂、免疫调节剂、单克隆抗体等新药及免疫治疗、自体造血干细胞移植应用于多发性骨髓瘤（MM），MM患者的预后和生存显著改善[1]–[2]。多药联合方案能提高新诊断MM（NDMM）患者移植前后的疗效。研究表明，三药方案与两药方案相比能够明显延长患者的无进展生存（PFS）时间[3]。自体造血干细胞移植后患者PFS时间更长[4]–[5]。来那度胺、硼替佐米和地塞米松（VRD）方案的广泛应用提高了诱导治疗的疗效，这一结论已在国外大型临床研究中得到证实[4],[6]–[8]。

近些年，VRD方案在真实世界治疗NDMM患者的安全性和疗效陆续报道，但国内的报告较少，涉及的病例数也仅有数十例[9]。自2018年开始，苏州大学附属第一医院在NDMM患者中进行了一项VRD方案联合自体造血干细胞移植或VRD方案持续治疗的登记性项目，本研究回顾性分析了截至2021年2月在本中心接受VRD方案治疗的150例NDMM患者的临床资料，评价VRD方案治疗NDMM的疗效和安全性。

## 病例与方法

1. 病例与治疗：纳入2018年11月至2021年2月在苏州大学附属第一医院诊断并接受VRD方案诱导治疗的150例NDMM患者。所有患者的诊断和治疗均符合中国多发性骨髓瘤诊治指南（2017版）[10]，美国东部肿瘤协作组（ECOG）评分0～2分。

所有患者在诱导治疗前完善的实验室检查包括血常规、生化常规（血清白蛋白、球蛋白、总蛋白、血钙、乳酸脱氢酶、肌酐）、β_2_-微球蛋白、血清蛋白电泳、免疫固定电泳、血清游离轻链、外周血形态分析、骨髓细胞形态学、免疫分型、荧光原位杂交（FISH）、骨髓活检；辅助检查：心电图、心脏超声、全身低剂量CT、DWI-MRI。遗传学高危定义为：FISH检测到t（4;14）、t（14;16）、t（14;20）、17p−。所有患者治疗前根据影像学评估是否合并髓外病灶，同时测量病灶大小。

标准的VRD方案为21 d一个疗程：硼替佐米1.3 mg·m^−2^·d^−1^，第1、4、8、11天，皮下注射；来那度胺25 mg/d，第1～14天，口服；地塞米松20 mg/d，第1、2、4、5、8、9、11、12天，静脉滴注或口服。肾功能不全患者根据肌酐清除率调整来那度胺剂量：肌酐清除率≥60 ml/min时，来那度胺25 mg/d；肌酐清除率≥30 ml/min但<60 ml/min时，来那度胺10 mg/d；肌酐清除率<30 ml/min及透析的患者，来那度胺10 mg/d，隔日1次。出现周围神经病变（PN）时，硼替佐米需减量：PN 1级不需要调整；PN 1级伴疼痛或2级，硼替佐米减至1.0 mg/m^2^，每周2次；PN 2级伴疼痛或3级，硼替佐米减至0.7 mg/m^2^，每周2次，或1.0 mg/m^2^，每周1次；出现PN 3级伴疼痛或4级时停用硼替佐米。体能状态差的患者地塞米松剂量减半；如治疗过程中出现感染，地塞米松剂量减半或停药。

应用甲钴胺预防周围神经病变，肾功能正常的患者使用唑来膦酸治疗骨病，肾功能不全的患者使用氯膦酸二钠治疗骨病。肾功能不全患者治疗过程中注意避免应用肾毒性药物，必要时可予透析治疗。完成4个疗程诱导化疗疗效达部分缓解（PR）及以上，适合移植且同意行移植的患者进行造血干细胞动员和采集，然后行自体造血干细胞移植，移植后进行巩固维持治疗；不适合移植或不考虑行移植的患者继续原方案治疗8个疗程后接受维持治疗。该研究获得苏州大学附属第一医院伦理委员会审查批准（2021伦研批第93号）。

2. 疗效评估：诱导治疗4个疗程后进行疗效评估。所有患者的疗效评估均根据国际骨髓瘤工作组（IMWG）制定的疗效判断标准[11]，疗效分为：疾病进展（PD）、疾病稳定（SD）、PR、非常好的部分缓解（VGPR）、完全缓解（CR）、严格意义的完全缓解（sCR）。诱导治疗结束后行十色流式细胞术监测微小残留病（MRD）水平。

3. 不良反应：不良反应的判定及分级根据美国国家癌症研究所不良事件常用术语标准（NCI-CTCAE）4.0版。

4. 随访：随访截止日期为2021年10月31日，中位随访时间22（1～38）个月，随访资料来源于住院、门诊病历及电话随访记录。

5. 统计学处理：采用SPSS 22软件进行数据分析，分类变量采用例数（百分比）表示，连续性变量采用均值或中位数（范围）表示。采用*t*检验、近似*t*检验、非参数秩和检验比较组间差异，率的比较采用*χ*^2^检验。生存曲线应用Graphpad Prism 8.0软件绘制，采用Log-rank检验比较生存差异。*P*<0.05为差异有统计学意义。

## 结果

1. 临床特征：纳入研究的患者共150例，男87例（58.0％），女63例（42.0％），中位年龄58（31～73）岁，120例（80.0％）患者年龄≤65岁，3例（2.0％）患者年龄>70岁；IgG型、IgA型、IgD型、轻链型患者分别为74例（49.3％）、30例（20.0％）、7例（4.7％）、39例（26.0％）。ISSⅠ期、Ⅱ期、Ⅲ期患者分别为26例（17.6％）、64例（43.2％）和58例（39.2％）。R-ISSⅠ期、Ⅱ期、Ⅲ期患者分别为19例（13.0％）、99例（67.8％）和28例（19.2％）。细胞遗传学高危［伴17p−、t（4;14）、t（14;16）、t（14;20）］患者38例（25.9％），肾功能不全（肌酐清除率<40 ml/min）患者48例（32.0％），12例（8.0％）患者初诊时合并髓外病灶。

2. 患者的治疗情况：150例患者中2例（1.3％）患者治疗早期死亡，148例（98.7％）完成诱导治疗。53例（35.3％）患者因不良事件药物减量；10例（6.7％）患者因不良事件停药，更换其他治疗方案。116例（77.3％）患者动员采集自体造血干细胞，其中5例行挽救性移植，3例患者动员失败，108例（72％）患者进行自体造血干细胞移植。至随访结束，进行移植的108例患者中11例出现疾病进展，5例死亡。未进行造血干细胞动员的32例患者及3例动员失败的患者中29例持续治疗，至随访结束，12例出现疾病进展，3例死亡；另外6例患者终止治疗，其中4例进展，2例死亡。

3. 疗效：138例患者可以评估疗效，2例在诱导治疗过程中早期死亡，10例在诱导结束后未评估疗效。总体反应率（ORR）为86.0％，CR/sCR率为54.4％，≥VGPR率为77.3％，诱导治疗结束后MRD阴性（<10^−4^）者69例（46.0％）；116例患者进行了造血干细胞动员及采集，采集物合格者101例（87.1％），其中采集物优秀者48例（41.4％）（优秀定义为采集物中CD34^+^细胞计数≥5×10^6^/kg，合格定义为采集物中CD34^+^细胞计数≥2×10^6^/kg）。遗传学高危和标危患者疗效和造血干细胞采集情况的差异均无统计学意义（*P*值分别为0.456和0.943）（表1）。

**表1:** 遗传学高危和标危多发性骨髓瘤患者诱导治疗后疗效和造血干细胞采集情况［例（％）］

疗效及造血干细胞采集	总体（150例）	高危患者（38例）	标危患者（109例）	*χ*^2^值	*P*值
疗效				4.681	0.456
CR或sCR	74（49.3）	23（60.5）	51（46.8）		
VGPR	42（28.0）	6（15.8）	33（30.3）		
PR	13（8.7）	3（7.9）	10（9.2）		
SD	1（0.7）	0（0）	1（0.9）		
PD	8（5.3）	3（7.9）	5（4.6）		
不可评估	12（8.0）	3（7.9）	9（8.3）		
≥VGPR	116（77.3）	29（76.3）	84（77.1）	0.009	0.925
ORR	129（86.0）	32（84.2）	94（86.2）	0.095	0.758
微小残留病					
阳性	69（46.0）	17（44.7）	49（45）		
阴性	69（46.0）	18（47.4）	51（46.8）	0.007	0.997
不可评估	12（8.0）	3（7.9）	9（8.3）		
造血干细胞采集情况^a^				0.386	0.943
优秀	48（41.4）	10（37.0）	38（42.7）		
合格	53（45.7）	13（48.1）	40（44.9）		
不合格	12（10.3）	3（11.1）	9（10.1）		
失败	3（2.6）	1（3.7）	2（2.2）		

注：CR：完全缓解；sCR：严格意义的完全缓解；VGPR：非常好的部分缓解；PR：部分缓解；SD：疾病稳定；PD：疾病进展；ORR：总体反应率；^a^共116例患者进行造血干细胞采集，其中高危患者27例，标危患者89例

在合并髓外病灶的12例患者中，1例未评估疗效，5例（41.7％）获得CR/sCR，4例（33.3％）获得PR，1例（8.3％）SD，1例（8.3％）PD。合并肾功能不全的患者中，42例可评估疗效，22例（45.8％）获得CR/sCR，14例（29.2％）获得VGPR，2例（4.2％）获得PR，4例（8.3％）PD。

4. 生存分析：16例（10.7％）患者死亡，32例（21.3％）患者疾病进展或复发，中位PFS和OS时间均未达到，3年PFS率为59％，3年OS率为83％。遗传学高危、标危患者的2年PFS率分别为65％和83％，2年OS率分别为77％和91％。遗传学高危患者的中位PFS时间较标危患者缩短（33个月对未达到，*P*＝0.014），中位OS时间的差异无统计学意义（均未达到，*P*＝0.072）（图1）。ISSⅠ期、Ⅱ期和Ⅲ期患者的2年PFS率分别为96％、77％、73％（*P*＝0.048）；2年OS率分别为100％、92％、81％（*P*＝0.064）（图2）。R-ISSⅠ期、Ⅱ期和Ⅲ期患者2年PFS率分别为100％、81％和54％（*P*＝0.002）；2年OS率分别为100％、90％和74％（*P*＝0.005）（图3）。

**图1:**
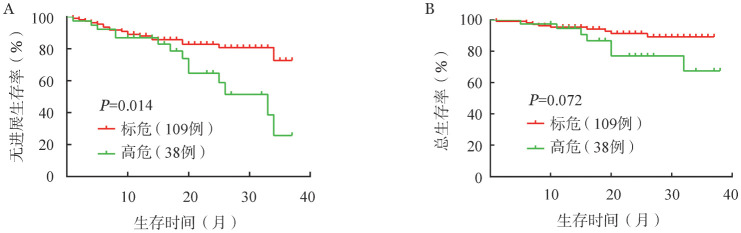
细胞遗传学高危和标危多发性骨髓瘤患者的无进展生存（A）和总生存（B）曲线

**图2:**
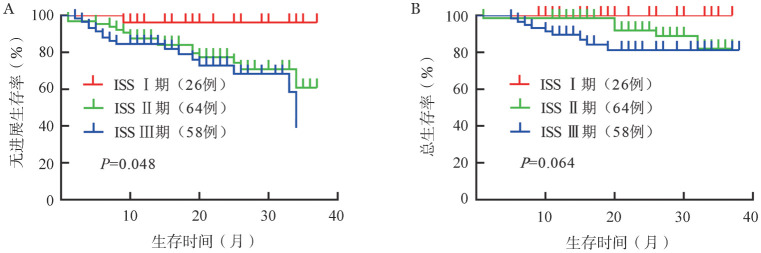
ISSⅠ期、Ⅱ期、Ⅲ期多发性骨髓瘤患者的无进展生存（A）和总生存（B）曲线

**图3:**
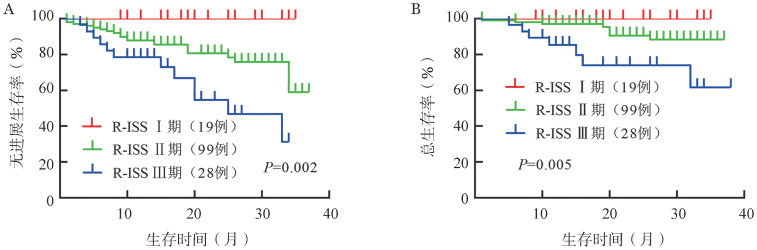
R-ISSⅠ期、Ⅱ期、Ⅲ期多发性骨髓瘤患者的无进展生存（A）和总生存（B）曲线

5. 安全性：诱导治疗期间血液学不良事件中血小板减少发生率最高（72％），其中3级和4级血小板减少发生率分别为16.0％和6.7％；中性粒细胞减少和贫血的发生率分别为42.0％和20.0％，其中3级发生率分别为8.7％和8.0％，无4级发生。非血液学不良事件中PN发生率最高（56.7％），其中PN 3级发生率为8.7％，无PN 4级发生。消化道反应主要为便秘、腹泻和肠梗阻，发生率分别为30.0％、17.3％和3.3％，均为1～2级；感染主要为上呼吸道感染、肺部感染和泌尿系统感染，发生率分别为23.3％、7.3％和4.7％，另有3.3％患者发生其他部位感染，其中3级上呼吸道感染、肺部感染、泌尿系统感染和其他部位感染的发生率分别为3.3％、5.3％、1.3％和3.3％，无4级感染发生。发热和肢体水肿发生率分别为10.0％和9.3％，无3级及以上。转氨酶升高和胆红素升高发生率分别为32.6％和5.3％，其中3级转氨酶升高发生率为3.3％，无3级及以上胆红素升高。皮疹、血栓、肾功能损伤和带状疱疹发生率分别为10.7％、2.7％、9.3％和2.0％，其中3级事件发生率分别为4.7％、1.3％、0.7％和0。48例肾功能不全患者的血液学不良事件发生率明显高于肾功能正常患者（表2）。在非血液学不良事件中，肾功能不全患者腹泻、肢体水肿、发热、血栓、肾功能恶化的发生率高于肾功能正常患者（表2）。

**表2:** 肾功能不全和肾功能正常多发性骨髓瘤患者应用VRD方案的安全性比较［例（％）］

不良事件	肾功能不全（48例）	肾功能正常（102例）	*χ*^2^值	*P*值
血小板减少	3（90.0）	65（63.7）	10.825	0.001
中性粒细胞减少	26（54.2）	37（36.3）	4.289	0.038
贫血	16（33.3）	14（13.7）	7.843	0.005
周围神经炎	23（47.9）	62（60.8）	2.201	0.138
消化道症状				
便秘	15（31.3）	30（29.4）	0.053	0.819
腹泻	13（27.1）	13（12.7）	4.683	0.030
肠梗阻	1（2.1）	4（3.9）	0.010	0.922
感染				
上呼吸道	10（20.8）	25（24.5）	0.247	0.619
肺部	5（10.4）	6（5.9）	0.433	0.511
其他	3（6.3）	2（1.9）	0.770	0.380
肢体水肿	10（20.8）	4（3.9）	9.124	0.003
发热	10（20.8）	5（4.9）	7.520	0.006
天冬氨酸转氨酶升高	12（25.0）	32（31.4）	0.639	0.424
丙氨酸转氨酶升高	11（22.9）	33（32.4）	1.402	0.236
胆红素升高	2（4.2）	6（5.9）	0.002	0.963
皮疹	3（6.3）	13（12.7）	1.445	0.229
血栓	4（8.3）	0（0）	5.817	0.016
肾功能恶化	10（20.8）	4（3.9）	9.124	0.003
带状疱疹	3（6.3）	0（0）	3.707	0.054

注：VRD方案：来那度胺+硼替佐米+地塞米松

## 讨论

在单中心150例NDMM患者的回顾性研究中，98％的患者年龄≤70岁，ECOG评分均为0～2分，患者一般情况较好，是移植的适用人群。但25.9％的患者遗传学高危，19.2％的患者R-ISS Ⅲ期，8％的患者初诊时合并髓外软组织病灶，超过30％的患者合并肾功能不全，高危患者比例较高。

本研究结果表明，VRD方案作为初诊患者的首选方案应答率高，但合并髓外病灶患者的疗效仍然较差。在SWOG SO777的研究中，接受8个疗程VRD方案治疗后，CR/sCR率为15.7％，ORR达81.2％[7]。在IFM2009研究中，未接受移植组的患者接受8个疗程VRD方案治疗，CR率达到49％，≥VGPR率达到78％，流式细胞术MRD阴性率65％[8]。国内贾静等[9]发表的研究显示，接受2个周期化疗，达到VGPR以上者占64.1％，接受4个周期化疗后，达到VGPR以上者占84.6％，遗传学高危与标危患者经VRD方案诱导治疗后≥VGPR率的差异无统计学意义（*P*＝0.952）。最近发表的一项回顾性研究纳入1 000例患者，显示VRD方案诱导治疗后的CR率为35.9％，≥VGPR率为67.6％，ORR为97.1％[12]。65岁及以上不适合移植的患者应用VRD-LITE方案（35 d为一个疗程；硼替佐米1.3 mg·m^−2^·d^−1^，第1、8、15、22天，皮下注射；来那度胺15 mg/d，第1～21天，口服；地塞米松20 mg/d，第1、2、8、9、15、16、22、23天，口服）也显示出良好的疗效，VRD-LITE方案的Ⅱ期临床研究显示，在中位年龄73岁的老年患者中应用VRD-LITE方案治疗4个疗程后，CR/sCR率为44％，≥VGPR率为66％，ORR为86％[13]。

本研究患者的中位PFS时间超过3年，3年OS率超过80％。国外一项纳入1 000例患者的回顾性研究报道，患者中位PFS时间为65个月，中位OS时间为126.6个月[12]。本研究中具有高危遗传学、R-ISS Ⅲ期、髓外软组织病灶、肾功能不全等高危特征患者占53.3％，可能会影响患者的整体预后。

本研究3级及以上血液学不良事件经暂停化疗药物、化疗药物减量、促造血治疗后均可恢复至< 1级，无血液学相关死亡事件发生。鉴于血小板低下发生率高，建议在发生3级血小板减少（PLT<50×10^9^/L）时停用抗凝药物，开始升血小板治疗，减少出血性事件发生，不延长治疗间隔。严重白细胞减少发生率低，除非患者有活动性感染病史，不建议常规进行抗生素预防和真菌预防，仅进行抗病毒预防。

PN 3级经营养神经、硼替佐米停药或减量后，大部分患者症状可缓解，少部分患者遗留比较严重的PN，需要长期停用硼替佐米，换用其他方案。鉴于PN发生率高，在治疗开始时常规预防性应用营养神经治疗，出现1级伴疼痛或2级及以上PN时及时调整硼替佐米剂量，在神经内科医师指导下积极开始专科治疗。2例患者在治疗过程中发生肺部感染死亡，其余3级感染事件经治疗后可恢复到<1级。3级丙氨酸转氨酶和天冬氨酸转氨酶升高患者经过保肝治疗后可以恢复至正常。其他少见的不良事件如肠梗阻、皮疹、带状疱疹的发生率均较低，经过治疗可恢复正常。

在SWOG SO777研究中，3级以上不良事件发生率高达82％，停药率达到20％，可能与静脉应用硼替佐米及每周两次给药相关。本研究3级及以上不良事件发生率43.4％，停药率6.7％，减药率35.3％，本研究中硼替佐米每周两次皮下给药，不良事件的发生率降低，尤其是3～4级。血栓事件的发生率均较低，可能是因为预防性使用抗凝药物，强调了全程抗凝药物使用的重要性。

初诊患者中合并肾功能不全的患者近1/3，肾功能不全患者血液学不良反应发生率高于肾功能正常的患者。可能是肾功能不全的患者来那度胺的代谢存在差异，导致体内药物浓度产生差异，即使在整个治疗过程中根据肾功能不全程度调整了来那度胺的剂量。在非血液学不良反应中，肾功能不全患者的肢体水肿、肾功能损害发生率高，主要是由于这部分患者肾脏基础功能差，水钠及代谢产物的排泄能力较差。肾功能不全患者血栓事件的发生率也较肾功能正常患者高，一方面可能是部分患者合并肾病综合征表现，处于相对高凝状态；另一方面，部分患者需要同时进行透析治疗，增加了血栓风险。需要特别注意的是，2例在治疗过程中因发生肺部感染死亡的患者均有肾功能不全。因此，治疗合并肾功能不全的NDMM患者时根据肌酐清除率调整VRD方案相对安全。

综上所述，VRD方案诱导治疗NDMM患者短期疗效显著，肾功能不全和高危患者的疗效也十分可观，但治疗过程中需要注意不良事件的管理，尤其是肾功能不全患者，尽可能减少停药率和减药率，使疗效最大化。然而本研究存在样本量较小、单中心回顾性研究等局限性，后期我们将开展多中心、前瞻性研究，扩大样本量，进一步探讨VRD方案治疗NDMM的疗效和安全性。
